# The impact of preconceptional obesity on trajectories of maternal lipids during gestation

**DOI:** 10.1038/srep29971

**Published:** 2016-07-20

**Authors:** Latife Bozkurt, Christian S. Göbl, Anna-Theresa Hörmayer, Anton Luger, Giovanni Pacini, Alexandra Kautzky-Willer

**Affiliations:** 1 Department of Internal Medicine III, Division of Endocrinology and Metabolism, Unit of Gender Medicine, Medical University of Vienna, Vienna, Austria; 2 Department of Obstetrics and Gynecology, Division of Feto-Maternal Medicine, Medical University of Vienna, Vienna, Austria; 3 Metabolic Unit, Institute of Neuroscience, National Research Council, Padova, Italy

## Abstract

Growing challenges of maternal obesity necessitate to focus metabolic management on alternative factors than glycaemia. The objective is to assess longitudinal changes in lipids and inflammatory parameters during pregnancies stratified by pregestational BMI. Therefore, 222 pregnant women (normal-weight BMI < 25: n = 91 (41%), overweight BMI 25–29.9: n = 69 (31%), obese BMI ≥ 30: n = 62 (28%)) underwent a detailed metabolic characterization including fasting lipids and glucometabolic parameters at <21^st^ gestational week (GW) with follow-up assessments at further three visits (24–28^th^ GW, 32–34^th^ GW, >36^th^ GW). Overweight and obesity was related to dyslipidemia already at baseline, i.e. elevated triglycerides (TG, p < 0.001), decreased high-density-lipoprotein-C (p = 0.009) and increased ultrasensitive-c-reactive-protein (usCRP, p < 0.001) independent of gestational diabetes prevalence. Trajectories of lipids during pregnancy progress revealed an unexpected less pronounced increase in TG, low-density-lipoprotein-C and total-cholesterol in overweight/obese women. usCRP remained associated with higher BMI throughout pregnancy showing no time-dependent longitudinal changes. Newborns of obese/overweight women were affected by higher birth-weight percentiles. Regarding lipids only maternal TG showed tendency for relation to prevalence of large-for-gestational-age offspring, particularly at the end of pregnancy (p = 0.048). Overweight and obese women show significant differences in trajectories of lipids during pregnancy that distinguish them from normal-weight women. Further studies should evaluate if targeting lipid metabolism could improve clinical management of maternal obesity.

Maternal obesity is accepted as another major risk factor that essentially impacts the whole pregnancy process and outcome^1^. There is increasing evidence that obesity is associated with a dysregulation in metabolic balance comprising lipid metabolism, inflammatory or hormonal processes in addition to insulin resistance^1^,^2^,^3^. Currently therapeutic management strategies in obese pregnancies are mainly focused on glycemic control since intrauterine hyperglycemia is causally relevant in the development of macrosomia, one of the most feared perinatal complications^4^. However, reaching optimal glucose targets in obese women is a challenging task due to the altered metabolic conditions and several studies show that despite improvement of glycemic control affected subjects still present increased morbidity compared to normal pregnancies^4^,^5^. Thus, also further aspects of maternal metabolism have to be issued in order to achieve a better pregnancy outcome.

Maternal lipid metabolism is affected by major changes that occur physiologically throughout gestation^2^. During first two trimester maternal hyperphagia and elevated insulin concentrations induce accumulation of fat depots by increasing fatty acid synthesis. Later in the last third of pregnancy lipoprotein levels increase along with higher lipoprotein triglyceride content (including increased low-density lipoproteins (LDL-C), high-density lipoproteins (HDL-C), very-low-density-lipoproteins (VLDL)) due to consecutively increasing insulin resistance and impact of pregnancy specific hormones^2^,^4^. There are only few studies evaluating longitudinal patterns of circulating lipids specifically in relation to maternal obesity. Reported conclusions are in part conflicting as there are substantial differences in methods e.g. variation in time of measurement, use of fasting or non-fasting samples and diagnostic criteria for gestational diabetes mellitus (GDM)^6^,^7^,^8^,^9^. However, basically previous results can be summarized by elevated triglycerides (TG) and lower high-density lipoproteins (HDL-C) as well as increased low-density lipoproteins (LDL-C) in obese women – a lipid profile indicating increased cardiovascular risk in diabetic populations^6^,^7^,^8^,^9^,^10^. Importantly, in this context maternal preconceptional obesity seems to exert greater sequelae than short-term weight changes, as excessive weight gain was not shown to affect the rate of change in lipid profiles in normal or overweight pregnancies^7^. Similarly, GDM pregnancies are characterized by alterations of pregnancy-driven changes in lipid metabolism but the extent to which obesity contributes to these associations is unclear^4^. Further, dyslipidemia in women with GDM history is regarded as surrogate marker for the manifestation of type 2 diabetes in later life^11^. Another condition that is suggested to principally accompany obesity is a state of low-grade chronic inflammation that may additionally trigger the manifestation of further secondary morbidities^12^. Also during pregnancy obesity driven inflammation might in part be implicated in the increased risk for both maternal as well as fetal complications^1^,^2^.

Therefore, this study aims to assess longitudinal patterns of metabolic parameters during pregnancy and their association with preconceptional overweight or obesity. A particular focus should be addressed on trajectories of serum lipids and subclinical inflammation as well as their relations with insulin resistance. Moreover, possible time-dependant associations of maternal lipids with offspring’s birth weights will be evaluated as further objective.

## Results

### Descriptive characteristics at baseline visit

Descriptive characteristics of the study population at first assessment (V1) grouped by preconceptional BMI categories are presented in Table 1. Accordingly, 91 (41%) women were classified as normal-weight, 69 (31%) as overweight and 62 (28%) as obese. Evaluation of glucose tolerance status during pregnancy revealed a higher rate of GDM manifestation in obese and overweight women. Measurements of glucose, insulin and C-peptide as well as estimates of insulin sensitivity (i.e. OGIS, QUICKI) at the early antenatal visit already showed significant differences between the subgroups. Insulin treatment was necessary in 42% (OR:3.97, 95%CI:1.88 to 8.69, p < 0.001) of obese and 30% (OR:2.24, 95%CI:1.05 to 4.94, p = 0.040) of overweight women compared to only 15% in the lean group.

Distinguishing results were observable in TG with increased values in obese and overweight women as well as in HDL-C, which in contrast tended to be significantly higher in lean subjects (Table 1). Further, TG (log-transformed) as well as HDL-C at baseline were significantly correlated with preconceptional BMI (TG: r = 0.32, p < 0.001, HDL-C: r = −0.18, p = 0.006) and with insulin sensitivity in terms of OGIS (TG: r = −0.31, p < 0.001, HDL-C: r = 0.18, p = 0.008). There were no differences for TC, LDL-C and NHDL-C observed between the groups. However, obese and overweight women showed higher concentrations of log-transformed usCRP compared to their normal-weight counterparts, which was shown to be inversely associated with insulin sensitivity (r = −0.26, p < 0.001). Differences between BMI categories remained unchanged after adjustment for GDM incidence.

### Longitudinal analysis of lipid parameters, sub-inflammation markers and weight

TG (log-transformed, ß:0.12, 95%CI:0.11 to 0.13, p < 0.001), TC (ß:11.5, 95%CI:10.3 to 12.8, p < 0.001), LDL-C (ß:6.6, 95%CI:5.6 to 7.6, p < 0.001) and HDL-C (ß:0.59, 95%CI:0.32 to 0.86, p < 0.001) showed an increase during pregnancy progress in the total study population, whereby ß describes the mean change by 4 weeks.

**Figure 1:**
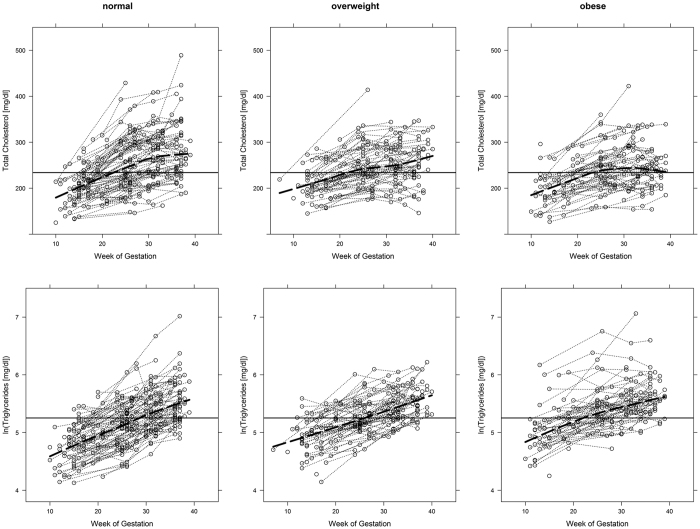
Trajectories of serum lipids and ultrasensitive C-reactive-protein during pregnancy categorized as preconceptional normal-weight, overweight or obese. Solid line represents the 50% quantile (i.e. the median) of all measurements during gestation. Dashed line represents a trend curve derived by locally weighted regression.

As compared to normal-weight women, females classified as obese and overweight show a less pronounced rise in TG (overweight: ß_group:time_:−0.03, 95%CI:−0.05 to −0.01, p = 0.002; obese: ß_group:time_:−0.05, 95%CI:−0.07 to −0.03, p < 0.001), LDL-C (overweight: ß_group:time_:−4.15, 95%CI:−6.52 to −1.78, p < 0.001; obese: ß_group:time_:−3.26, 95%CI:−5.68 to −0.85, p = 0.008) and TC (overweight: ß_group:time_:−5.52, 95%CI:−8.31 to −2.72, p < 0.001; obese: ß_group:time_:−3.73, 95%CI:−6.58 to −0.88, p = 0.011) during gestation. Trajectories of TG and TC are visualized in Fig. 1. However, no group-dependent differences in longitudinal patterns were observed for HDL-C: overweight vs. normal-weight: p = 0.979; obese vs. normal-weight: p = 0.069 (Table 1). Notably, usCRP remained higher in obese and overweight women until end of pregnancy with no time-dependent longitudinal changes. Moreover, mean increase in maternal weight during pregnancy tended to be higher in normal-weight females, but the difference was only significant compared to obese women (ß_group:time_:−0.79, 95%CI:−1.10 to −0.48, p < 0.001 i.e. changes in weight (kg) obese vs. normal).

Neither an adjustment for weight gain, nor for OGIS or GDM status changed our basic conclusions about the specific patterns of lipids or subclinical inflammation in overweight or obese mothers. In addition, we repeated our analysis after exclusion of subjects with no available data at the last (fourth) visit to exclude a possible impact of non-random dropouts. However, our basic conclusions on lipid trajectories remained unchanged.

### Trimester-specific association of lipid status with macrosomia

Percentiles of birth weight (available in 187 singleton pregnancies) based on international anthropometric standards^13^ were associated with maternal grade of BMI showing higher values with increasing grade of BMI (OB: 70.8 ± 27.3, p = 0.014; OW: 70.4 ± 25.2, p = 0.013 vs. NW: 58.7 ± 28.0). Among these newborns a total of 45 (24%) were identified as LGA (birth weight above the 90th percentile). Analysis of effects of lipids on birth weight outcome by visit showed no significant impact of LDL, HDL or Non-HDL at any time point. However, maternal TG (log-transformed) tended to be associated with LGA offspring, whereby this difference showed marginal significance by the end of pregnancy (visit 3: OR:3.25, 95%CI:0.97 to 11.42, p = 0.056, visit 4: OR:3.84, 95%CI:1.01 to 15.61, p = 0.048). However, preconceptional BMI was not associated with LGA although sample size has to be considered as a limitation.

## Discussion

Overweight and obese women show significant differences in trajectories of lipids during pregnancy that distinguish them from normal-weight women. Particularly, during early pregnancy obesity was associated with unexpected high levels of triglycerides and decreased HDL concentrations. Moreover, throughout gestation grade of longitudinal changes in serum lipids was lower in obese and overweight compared to normal-weight pregnant women.

In general, it is suggested that during course of pregnancy specific modulations in metabolism occur physiologically in order to sufficiently provide fuels for fetal development^4^. Changes in lipid metabolism that occur in this context are indicated to comprise: 1) an anabolic phase with accumulation of fatty acid in maternal depots in the first trimester as preparative measure for the 2) later catabolic state that emerges due to development of insulin resistance whereby the induction of peripheral adipose tissue lipolysis results in a normal rise in plasma triacylglycerols during last third of gestation^2^. According to this an increase in all plasma lipids during pregnancy progress was also observable in our study population.

However, examinations of mothers with metabolic complications show significant deviations from the normal pattern of lipid changes^4^,^14^,^15^. The focus of previous research was predominantly directed on pregnancies with diabetes but obesity was outlined as a main interacting factor in these analyses. Similarly to our results, expectant mothers with type-1 diabetes and coexistent metabolic syndrome were shown to present significantly higher triglycerides and lower HDL-C in first trimester along with higher weight indices compared to those prediabetic women without any other metabolic problems^14^.

Only a few studies selectively payed attention on the differential aspects of maternal obesity in relation to lipid metabolism. Meyer *et al*. reported higher values for triglycerides in the first trimester and a lower rate of increase during later pregnancy^8^, what is in accordance with our results although they examined a rather small sample size. Other longitudinal studies analyzing lipid data in association to obesity during pregnancy reported diverging results. Scifres *et al*. examined a mixed group comprising overweight and obese women twice at early and late pregnancy but at non-fasting state and showed a lower rate of change solely for TC and LDL-C^7^. Similarly, two other longitudinal studies reported higher baseline levels for triglycerides, TC and LDL-C. However, they indicated a less pronounced increase only for TC and LDL-C while an attenuation in triglycerides during pregnancy progress in overweight and obese pregnancies compared to the reference group was additionally observed in our study^6^,^9^. It is assumed that excessive triglycerides serve as substrate for the formation of small-dense LDL particles, which are regarded as an important atherogenic risk factor as they exhibit enhanced oxidation potential^8^. Sattar *et al*. concluded in their study comprising longitudinal data of 10 normal pregnancies that the amount of triglycerides in early gestation or the rate of increment may promote a shift to small-dense LDL during later gestation^16^. Accordingly, Meyer *et al*. also found higher proportions of small-dense LDL fractions in obese and overweight subjects^8^. Despite these conflicting results, we observed that as pregnancy progressed trajectories in obese women showed lower rates of changes in triglycerides as well as LDL-C and TC levels equally. These processes seem to be triggered by obesity as there were no particular differences in lipids during pregnancy course in terms of GDM status, insulin resistance or weight gain. In accordance to prior suggestions the significant correlation of baseline TG with degree of obesity and insulin resistance in earlier pregnancy might indeed be interpreted as precursory to the later development of adverse changes in lipids. Moreover, obese and overweight women exhibited lower HDL-levels, another commonly evidenced indicator of an unfavorable lipid constellation^10^. This condition in obese women is further accompanied by significantly higher levels of usCRP persisting during pregnancy.

Taken together, we hypothesize that unphysiologic hypertriglyceridemia at the beginning of pregnancy precipitated by pregestational obesity and insulin resistance might further derange normal lipid metabolism in a way that induces an inappropriate utilization of the excessive TG in e.g. increased very-low-density lipoprotein (VLDL) and small-dense LDL formation or ectopic fat accumulation especially in the liver what impacts the activity of lipase and additionally deteriorates the lipid profile in obese pregnancies. Further, a disarrangement of TG concentrations seems to impact the flexibility in the maternal adaptation to pregnancy related changes^8^. The complicating impact of obesity and insulin resistance is concurrently reflected in chronic subinflammation and in the development of endothelial dysfunction what is implicated in adverse outcomes going beyond pregnancy duration^17^.

However, the way how maternal obesity and eventually pregnancy-induced dyslipidemia developing normally in the last trimester concretely impact availability of substrates to the fetus and in which degree enhance fetal macrosomia is not completely understood. There is strong evidence, in particular by the data of the HAPO study, that hyperglycemia is associated with the risk for birth weight above the 90^th^ percentile^18^. However, macrosomia was also prevalent in offspring of pregestational type-1-diabetes as well GDM women with satisfactory glycemic control during pregnancy^4^,^5^. Hence other factors particularly maternal obesity and lipids attracted attention as potential intermediators that contribute to the persisting prevalence of macrosomia. In our previous study we already showed a significant association of triglyceride levels with abnormal fetal growth in pregnancies affected by type 1 and type 2 diabetes compared to uncomplicated pregnancies similarly to previously reported results for well-controlled GDM^15^. Langer *et al*. demonstrated that pregestational obesity significantly increases the risk for macrosomia in well-controlled GDM pregnancies. Of note, the rate of macrosomia in obese women with GDM was lowered in cases receiving insulin therapy compared to the normal-weight reference group^19^. This effect of insulin therapy may be due to the reduction of lipolysis by insulin action^19^. Apart from GDM, in pregnancies with normal glucose tolerance it is already recognized that offspring of obese and overweight show higher risk for macrosomia compared to those of lean women apparently due to increase in neonatal fat and not lean body mass^20^. Elevated triglycerides as it is the case in obese and insulin resistant women, increase the availability of free fatty acids for the fetus via hydralization by placental lipoprotein lipase and enhanced placental transport^21^. Indicative for this theory, in our study population neonatal birth weight percentiles were increased in obese and overweight women, who compared to lean pregnant mothers also showed a dyslipidemic profile in particular elevated triglycerides during pregnancy course. Actually, LGA prevalence became marginally associated with elevated triglycerides with progressing pregnancy. However, our sample size with low numbers of LGA cases has to be considered.

In summary, our study results imply that obesity during gestation is associated with an abnormal lipid constellation characterized by significant hypertriglyceridemia and low HDL-C at earlier pregnancy that persists as pregnancy proceeds. Moreover, we observed distinguishing patterns of LDL-C and TC trajectories as well as a higher degree of subclinical inflammation (in terms of usCRP) in relation to lean women. With regard to fetal outcome, we suggest a possible causal contribution of maternal lipids on macrosomia as adverse neonatal weight outcome; concretely, prevalence of large-for-gestational-age offspring tended to be positively related to triglycerides particularly by the end of pregnancy. The debate whether obesity or rather diabetes during pregnancy have more detrimental effects on pregnancy outcome will remain inconclusive as long their impact on other pathophysiologic aspects as well as their causalities are not determined. Given the apparent deviations in lipids that are significantly associated with degree of maternal obesity, optimization of lipid metabolism should be considered as another measure in routine practice for the improvement of clinical outcomes.

## Methods

### Study population

This prospective longitudinal study was performed at the Division of Endocrinology and Metabolism, Department of Internal Medicine III, and Division of Feto-Maternal Medicine, Department of Obstetrics and Gynecology, Medical University of Vienna between 2010–2014. Study participants attending our diabetes and pregnancy outpatient clinics at a tertiary care center were recruited at ≤21^st^ week of gestation by preliminary excluding women with chronic or serious acute infections, hematological diseases or diseases of the hematopoietic system, severely impaired liver or kidney function or infectious diseases. After consent of identified eligible women enrolment in study and assessment of glucose tolerance was performed <21^st^ GW (visit 1). During this first assessment four women were classified as having overt diabetes (i.e. fasting plasma glucose 126 mg/dl, HbA1c > 6.5% (47.54 mmol/mol)) and hence were excluded from further participation. Further clinical evaluations followed until delivery at 24^th^–28^th^ GW (visit 2), 30^th^–34^th^ GW (visit 3) and >36^th^ GW (visit 4). The study was approved by the local ethics committee (Ethics Committee of the Medical University of Vienna) and was performed in accordance with the Declaration of Helsinki. All subjects gave written informed consent for participation in the study.

### Clinical examinations and follow-ups

All women underwent a broad risk evaluation at initial assignment (≤21^st^ GW) including BMI (preconceptional and actual), age, obstetric history. The calculation of preconceptional BMI was based on the self-reported indication of pregestational weight by the pregnant women as also applied by several actual studies related to this topic^6^,^7^,^9^ and in accordance with validation analyses showing high correlation with measured weight during pregnancy^22^ as well as in samples of non-pregnant young women^23^. Moreover, we observed a high correlation between self-reported pregestational weight and measured weight at the first visit, early in pregnancy (r = 0.97). A 2 h-OGTT was performed at V1 (median: 16 weeks, IQR: 14–18) after a 10–12 hour overnight fast. Determination of glucose, insulin and C-peptide was performed in venous blood samples drawn at fasting as well as 30, 60, 90 and 120 min following a 75 g-glucose load. A second clinical evaluation was conducted during 24–28^th^ week of pregnancy, whereby women with previous negative GDM examination result obtained a second diagnostic OGTT. Six subjects with negative OGTT results received insulin therapy during follow-up due to incident macrosomia and elevated fasting glucose and thus were classified as GDM. Details regarding glucometabolic state at baseline were previously described^24^. Hence, after exclusion of four women with preexisting diabetes a total of 222 women were subdivided into three groups according to the calculated preconceptional BMI: normal-weight with BMI < 25 (NW), overweight with BMI 25–29.9 (OW), obese with BMI > 30 (OB) as established by the World Health Organization^25^. At each visit a venous blood sample was drawn in fasting condition for further laboratory analysis.

### Laboratory measurements

In addition to glucometabolic parameters, serum TG, total-cholesterol (TC), HDL-C, LDL-C and ultrasensitive C-reactive protein (usCRP) concentrations were evaluated at each visit. Glucose, insulin and C-peptide were determined from blood samples obtained during the OGTT as well as further visits. All parameters were measured according to the international standard laboratory methods at our certified Department of Medical and Chemical Laboratory Diagnostics ( http://www.kimcl.at/).

Insulin sensitivity was described by the insulin sensitivity index (OGIS), which represents insulin mediated glucose clearance during an OGTT and has been validated versus the glucose clamp^26^. Insulin sensitivity in fasting conditions was described by QUICKI^27^.

### Neonatal care and anthropometric

Immediately after delivery, birth weight was measured with a calibrated scale and birth length was determined to the nearest 0,1 cm using an infant board with a stadiometer. Calculations of age and sex adjusted percentiles were performed by using recently published international anthropometric standards^13^.

### Statistical analysis

Continuous variables were summarized by means and standard deviations (SD), and categorical variables by counts and percentages. Comparisons of continuous parameters (cross-sectional) were performed by analysis of variance (ANOVA) and Fisher protected LSD tests for group-specific differences. Differences of categorical variables were assessed by using Fisher´s exact test. Odds ratios and 95% confidence intervals (CI) were computed by logistic regression. The relationship between metric scaled variables (e.g birth weight, serum lipids and usCRP) at different visits was assessed by linear regression for multivariate analysis. Data transformations were performed if skewed distribution was detected within descriptive analysis: ln[x] for TG, and ln[x + 1] for insulin and usCRP. Linear mixed effects (LME) models with random intercepts and random slopes by subjects were used to assess time dependent changes of parameters of interest during follow-up in different groups. Longitudinal analyses by LME have the advantage of integrating all available data without completely excluding those with missing visits. For these analyses, regression coefficients (β) represent the mean change in the dependent variable for the increase of 4 weeks of gestation. A group per time interaction was included to assess group specific differences in time-dependent changes. There was no further specification of the covariance structure, as a continuous autoregressive process did not substantially improve our models. Statistical analysis was performed with R (V3.1.1) and contributing packages (particularly “gmodels”, “nlme”, “lattice”, and “beeswarm”)^28^. A two-sided p-value ≤ 0.05 was considered statistically significant. There were no considerations to adjust for multiplicity in this report if not otherwise indicated.

## Additional Information

**How to cite this article**: Bozkurt, L. *et al*. The impact of preconceptional obesity on trajectories of maternal lipids during gestation. *Sci. Rep.* **6**, 29971; doi: 10.1038/srep29971 (2016).

## Figures and Tables

**Table 1:** Characteristics and metabolic changes during pregnancy in relation to preconceptional weight (OB: obese, OW: overweight, NW: normal-weight).

	**N**	**NW**	**n**	**OW**	**n**	**OB**	**P**_**ANOVA**_	**P**_**group:time**_
Age (years)	91	32.7 ± 5.4	69	32.2 ± 4.9	62	30.9 ± 5.0	0.093	
Gestational week of first testing	91	16.1 ± 2.8	69	15.9 ± 3.0	62	15.5 ± 3.2	0.435	
GDM prevalence	91	23 (25.2%)	69	27 (39.1%)	62	34 (54.8%)§	0.001	
Insulin treatment	91	14 (15.4%)	69	20 (30.0%)	62	26 (41.9%)§	0.001	
FPG (mg/dl)	90	77.5 ± 6.0	69	81.1 ± 9.0§	62	85.1 ± 9.5*^,§^	<0.001	
Insulin (μU/ml)‡	91	1.5 ± 0.48	68	1.8 ± 0.72§	62	2.1 ± 0.73*^,§^	<0.001	
C-peptide (ng/ml)	90	1.2 ± 0.41	68	1.9 ± 1.35§	62	2.2 ± 0.89*^,§^	<0.001	
QUICKI	90	0.42 ± 0.04	68	0.40 ± 0.05§	62	0.37 ± 0.05*^,§^	<0.001	
OGIS ml/min/m^2^	78	490.2 ± 52.7	64	458.2 ± 61.5§	60	417.2 ± 66.9*^,§^	<0.001	
Pregestational BMI (kg/m^2^)	91	21.7 ± 2.1	69	27.0 ± 1.3§	62	34.3 ± 4.4*^,§^	<0.001	
BMI (kg/m^2^) V1	91	23.5 ± 2.6	69	28.4 ± 1.7§	62	35.5 ± 4.8*^,§^	<0.001	<0.001
BMI (kg/m^2^) V2	83	25.4 ± 2.6	59	30.4 ± 2.1§	53	36.8 ± 5.0*^,§^	<0.001	
BMI (kg/m^2^) V3	65	26.6 ± 2.7	52	31.3 ± 2.3§	46	37.5 ± 5.3*^,§^	<0.001	
BMI (kg/m^2^) V4	58	27.3 ± 2.8	39	31.9 ± 2.0§	36	37.9 ± 5.7*^,§^	<0.001	
Triglycerides (mg/dl) V1†	90	4.82 ± 0.34	69	4.97 ± 0.32§	62	5.10 ± 0.39*^,§^	<0.001	<0.001
Triglycerides (mg/dl) V2†	82	5.15 ± 0.38	59	5.26 ± 0.31	51	5.44 ± 0.39*^,§^	<0.001	
Triglycerides (mg/dl) V3†	66	5.38 ± 0.36	52	5.43 ± 0.28	48	5.55 ± 0.41§	0.035	
Triglycerides (mg/dl) V4†	38	5.55 ± 0.40	42	5.58 ± 0.28	56	5.63 ± 0.30	0.517	
Total cholesterol (mg/dl) V1	90	208.2 ± 40.4	69	216.1 ± 35.6	62	208.9 ± 41.5	0.410	<0.001
Total cholesterol (mg/dl) V2	82	254.1 ± 59.4	59	247.6 ± 46.6	51	243.8 ± 48.2	0.528	
Total cholesterol (mg/dl) V3	66	272.7 ± 55.4	52	252.8 ± 44.3§	48	251.4 ± 55.5§	0.048	
Total cholesterol (mg/dl) V4	56	280.9 ± 61.7	42	259.3 ± 48.6§	38	241.7 ± 40.6§	0.002	
LDL-cholesterol (mg/dl) V1	90	113.6 ± 34.3	69	122.1 ± 29.1	62	115.8 ± 34.4	0.261	0.001
LDL-cholesterol (mg/dl) V2	81	144.4 ± 50.0	59	138.8 ± 38.6	51	133.3 ± 42.3	0.378	
LDL-cholesterol (mg/dl) V3	66	155.9 ± 46.2	52	140.1 ± 38.4	47	136.2 ± 49.8§	0.047	
LDL-cholesterol (mg/dl) V4	55	156.9 ± 45.5	40	142.7 ± 42.6	38	124.9 ± 36.8§	0.002	
HDL-cholesterol (mg/dl) V1	90	68.2 ± 13.1	69	63.9 ± 14.8§	62	61.7 ± 11.2§	0.009	0.132
HDL-cholesterol (mg/dl) V2	81	73.9 ± 15.7	59	70.0 ± 18.1	51	68.3 ± 12.8	0.109	
HDL-cholesterol (mg/dl) V3	66	72.3 ± 16.1	52	66.1 ± 17.4§	47	65.8 ± 10.5§	0.034	
HDL-cholesterol (mg/dl) V4	55	70.4 ± 14.3	40	64.5 ± 17.4	38	65.8 ± 11.7	0.116	
NHDL-cholesterol (mg/dl) V1	90	139.9 ± 39.1	69	152.2 ± 32.7	62	147.2 ± 36.7	0.106	<0.001
NHDL-cholesterol (mg/dl) V2	81	179.3 ± 55.7	59	177.6 ± 41.9	51	175.5 ± 44.2	0.912	
NHDL-cholesterol (mg/dl) V3	66	200.4 ± 52.6	52	186.7 ± 41.5	47	183.8 ± 52.3	0.154	
NHDL-cholesterol (mg/dl) V4	55	206.7 ± 55.2	40	193.6 ± 41.5	38	175.9 ± 38.3§	0.009	
usCRP (mg/dl) V1‡	85	0.38 ± 0.28	67	0.50 ± 0.32§	62	0.69 ± 0.28*^,§^	<0.001	0.376
usCRP (mg/dl) V2‡	51	0.34 ± 0.22	81	0.47 ± 0.28§	51	0.72 ± 0.37*^,§^	<0.001	
usCRP (mg/dl) V3‡	63	0.34 ± 0.19	52	0.48 ± 0.32§	48	0.56 ± 0.26§	<0.001	
usCRP (mg/dl) V4‡	55	0.32 ± 0.24	38	0.39 ± 0.31§	37	0.59 ± 0.28§	<0.001	

Data represent means and standard deviations as well as count and percentages. FPG (fasting plasma glucose), QUICKI (quantitative insulin sensitivity check index), OGIS (oral glucose insulin sensitivity index), BMI (body mass index), LDL (low density lipoprotein), HDL (high density lipoprotein), NHDL (non high density lipoprotein), usCRP (ultrasensitive C-reactive protein).

^†^Transformation ln[x].

^‡^Transformation: ln[x + 1]. P_ANOVA_ were determined by one-way analysis of variance at each visit.

^§^vs. NW.

^*^vs. OW. P_group:time_ determined by mixed-effects models (likelihood ratio test) refers to the global null hypothesis that the time related change of all three groups is comparable.
